# Four decades of Indian Journal of Pharmacology: An overview

**DOI:** 10.4103/0253-7613.42299

**Published:** 2008-06

**Authors:** Varsha Patel

**Affiliations:** Department of Pharmacology, Smt. NHL Municipal Medical College, Ahmedabad, India. E-mail: ijp@synchronresearch.com

Indian Journal of Pharmacology (IJP) will step into the fifth decade in 2009. It is customary to celebrate ‘jubilee’ years with some sense of gratification and achievement; a time for looking back to take the account of progress made along with a note of milestones that are yet to be achieved. The next introspective analysis may be taken up in 2018-19, the golden jubilee year of IJP.

However, retrospection and overview of IJP at the end of the fourth decade could also be a worthwhile exercise. Why? Firstly, IJP has seen rapid changes in the last decade from slow ‘hard’ print form to fast ‘soft’ online version and many more. Secondly, four decades is a period long enough for an overview. It would also provide a nostalgic plunge for many who have contributed to sustenance and progress of this extract of research in the field of pharmacology in India as editors, reviewers or authors. Lastly, for young and budding pharmacologists it will be like peeping in the glorious past of pharmacology research in India. Following is the essence of a journey down the lanes of IJP Archives CD 2001 and across ijp-online.com from year 1969 onwards going through the contents of each issue and the articles, wherever appropriate.

The year 1969 will always be remembered with pride by all of us associated with pharmacology. This was the year when Indian Pharmacological Society (IPS) was born to enjoy an independent existence, rather than as conjoint twin brother (or sister) of physiology since 1955. It was the outcome of great vision and wisdom of stalwarts in pharmacology like Professors K.P. Bhargava, U.K. Sheth, P.C. Dandiya, M.L. Gujaral, M.N. Ghosh, A.K. Sanyal, K.C. Singhal and others to start an official publication of the society, Indian Journal of Pharmacology, also in the same year. In his first editorial Prof. K.P. Bhargava wrote “do not forget you are the society and your mouthpiece is your journal.” Since then a continuous march forward is on for four decades and shall continue further for decades to come. Some of the pioneers are not with us today but they have left behind their footprints for the present and future generations of pharmacologists to follow and proceed ahead.

The inaugural issue contained editorials along with reviews on various aspects of pharmacology from ancient pharmacology to its development as a modern science, drug development etc. From the second volume (1970) onwards, research papers, focusing more on experimental pharmacology- pertaining to central nervous system, psychopharmacology, indigenous drugs, cardiovascular system, autacoids, NSAIDS were published. The first clinical trial published was ‘Glycerrhiza glabra in rheumatoid arthritis.’ Reports on symposia, review articles, and even special issues helped in updating the readers with current areas of research. The last year saw change in editor with further changes in the journal.

The second decade (1979-88) saw more of clinical pharmacology in the form of clinical trials, bioavailability studies, pharmacokinetic studies and drug interactions. In the same way presence of histamine and autonomic pharmacology was felt. Research papers also covered some diverse topics while exploring receptor pharmacology and newer actions of established drugs.

The third decade (1989-98) saw reports on experimental methods, clinical trials, hormone related research, ADR reports, drug utilization studies, therapeutic drug monitoring, calcium channel blockers, beta-2 agonists, drug interactions, drug kinetics, bioavailability and bioequivalence. Reports on use of computers in research appeared from time to time. The readers were updated by large numbers of minireviews and reviews. Year 1993 marked the silver jubilee year of IPS and IJP.

The fourth decade (1999-2008) welcomed the new millennium with a special article on  “Evolution of pharmacology in India during twentieth century”. The readers were made aware of CPCSEA rules for breeding and conducting animal experiments. Research publications included more of indigenous drugs, along with drug kinetics, newer actions of established drugs, clinical pharmacology including drug use studies, case reports on ADR and ADR monitoring. This decade is remarkable due to several changes brought about by the editors, for the improvement of the standard of IJP.

“It is said that ---- a society is known by the journal it publishes”, Professor Bhargava wrote in his editorial of the inaugural issue. Over four decades the journal has been driven by the editors who have strived to elevate its status and credentials among pharmacology fraternity. From Prof. K.P. Bhargava (1969-1971), the other editors who were at the helm of IJP are Prof. U.K. Sheth (1972-1974), Prof. P.C. Dandiya (1975-1977), Prof. M.N. Ghosh (1978-1980), Prof. A.K. Sanyal (1981-1983), Prof. K.C. Singhal (1989-1991), Prof. C. Adithan (1992-2000), Prof. R. Raveendran (2001-2006) and Dr. Shivprakash (2007 onwards).

The cover page has seen cosmetic changes from plain white, shades of blue and green, occasional red to double colored to getting more and more colourful from 2001 onwards and with attractive graphics at present, thanks to advances in printing technology. The IPS emblem appeared from 1972. Desktop publication and offset printing was introduced from 1992, when journal started accepting articles on floppies. From a quarterly journal in the first two decades, IJP became bimonthly in 1997. The editor Prof C. Adithan also made other changes in format and publication policy like standard IMRAD format with structured abstract, use of recent references, greater use of letter to editor section and introduction of fillers. Later on IJP became more hi-tech with submission on CD or by E-mail being introduced. From year 2001 onwards, Prof Raveendran brought further changes regular editorials on issues mainly related to scientific writing. With ‘e’ and ‘www’ fast becoming the catch words, IJP website was launched. IJP was available online since 1999, being one of the few Indian journals to go like that. IJP's indexing with SCI was another major achievement in the last decade.

Over four decades, the journal has seen more and more variety being added to its platter. Frequency of articles increased with minireviews from 1978 onwards. The section on postgraduate pharmacology was introduced from 17^th^  volume, (1985) with more editorials including guest editorials. From 1990 (Vol.22) references changed from Harvard style to Vancouver format to meet the international requirements. From 1993 the section on educational forum replaced PG pharmacology section. From 1994 onwards book reviews and forthcoming events were seen more frequently while section on software review started from 1998. The first ever compilation of all issues from first volume (1969) to vol. 32 (2000) in form of a CD was released at IPS conference of 2000. We are greatly indebted to the editorial team from JIPMER Pondicherry, under the leadership of Prof. C.Adithan for this Herculean task. Updated versions of IJP archives CD was released every year thereafter. From 2001, further features like ‘molecules of millennium’ and ‘webwise’ were added. Both kept the readers updated regarding new molecules on horizon and websites useful for pharmacologist fraternity. The present editorial team is committed to take IJP forward; a special supplement on ‘Pharmacovigilance’ is an example.

Pharmacology education in medical colleges, both undergraduate and postgraduate has attracted attention throughout the last forty years and is the subject for reviews, viewpoints and reports of new curricular changes introduced at different institutes. Research papers on indigenous drugs draw attention by their predominant presence throughout four decades but more so in the last decade. The probable reasons are out of scope of this article.

The share of contribution from medical colleges, pharmacy institutions, research institutes and others like pharmaceutical companies have changed over time. Contributions from pharmacy institutes have increased over time while contributions from national research institutes, veterinary institutes, botanical and agricultural institutes and pharmaceutical companies continue to appear sporadically. The journal has also attracted scientists from European countries, neighboring Asian countries, African countries and from Middle East in the form of special articles, review articles or research papers from time to time.

This was a journey down the memory lane and back in the form of overview of four decades of IJP. The task would have been impossible without referring to IJP-archives CD-2001 and online issues from 2002 onwards. As it is an overview, omissions if any are unintentional. IJP has come a long way and has achieved a respectable status in being one of the indexed journals from India in the field of medicine. However a lot needs to be done. Somehow, a feeling that Indian journals including IJP are treated as ‘Trash-cans’ by some if not all the contributors, as voiced in his first editorial in 2001 by Prof. Raveendran still persists. Let us contribute our best scientific work to the journal and make the Golden Jubilee year more satisfying for the editors as well as the readers..

